# Butyrate Increases Heparin Synthesis and Storage in Human Mast Cells

**DOI:** 10.3390/cells13151241

**Published:** 2024-07-24

**Authors:** Syed Benazir Alam, Zhimin Yan, Nishita Hiresha Verma, Larry D. Unsworth, Marianna Kulka

**Affiliations:** 1 Quantum and Nanotechnologies Research Centre, National Research Council Canada, Edmonton, AB T6G 2M9, Canada; syedbenazir.alam@nrc-cnrc.gc.ca (S.B.A.); zhimin.yan@nrc-cnrc.gc.ca (Z.Y.); nishitah@ualberta.ca (N.H.V.); 2 Department of Chemical and Materials Engineering, University of Alberta, Edmonton, AB T6G 1H9, Canada; lunswort@ualberta.ca; 3 Department of Biomedical Engineering, University of Alberta, Edmonton, AB T6G 1H9, Canada; 4 Department of Medical Microbiology and Immunology, University of Alberta, Edmonton, AB T6G 2E1, Canada

**Keywords:** butyrate, mast cells, sulfated glycosaminoglycan, heparin, berberine

## Abstract

Sulphated glycosaminoglycans (GAGs) such as heparin are a major component of mast cell granules and form the matrix within which biogenic mediators are stored. Since GAGs released from mast cells also play an important role in helminth expulsion, understanding GAG storage can offer new insights into mast cell function. Sodium butyrate (NaBu), a short-chain fatty acid, causes ultrastructural changes within the granules of human mast cells (HMC-1) and increases their histamine content. Therefore, we hypothesized that NaBu treatment would also modify the storage of polysaccharides such as GAGs. NaBu (1 mM) significantly increased GAG content and granularity in a time- and concentration-dependent manner without affecting cell viability and metabolic activity. NaBu increased the expression of enzymes associated with heparin biosynthesis (*GLCE*, *NDST1*, *NDST2*, *HS6ST1*, and *GALT1*) in a time-dependent manner. A cholesteryl butyrate emulsion (CholButE) increased heparin content after 24 and 48 h and modestly altered the expression of genes involved in heparin biosynthesis. Similar to NaBu, CholButE reduced cell proliferation without significantly altering viability or metabolic activity. These data show that butyrate increases the synthesis and storage of heparin in human mast cells, perhaps by altering their metabolic pathways.

## 1. Introduction

Butyrate is a short-chain fatty acid that is produced by the fermentation of non-digestible dietary fiber by intestinal microbiota and is an important regulator of gut health. Although butyrate and other short-chain fatty acids are important mediators in the gastrointestinal tract, they are also found at lower levels in the blood and can reach the lungs and skin via circulation [1,2]. Butyrate is a primary source of energy for some intestinal cells, including colonocytes [3], and functions as a histone deacetylase inhibitor (HDACi) [4]. Since butyrate favors histone acetylation, it promotes an open and transcriptionally accessible state, allowing for the expression of otherwise quiescent genes [4]. Butyrate, like other short-chain fatty acids, mediates the effects of the gut microbiome on local and systemic immunity.

Located beneath the epithelial layer in the submucosa, mast cells are important immune cells in the gut. They are involved in regulating important physiological responses in the gut during helminth expulsion, gastro-inflammation, colitis, and inflammatory bowel disease [5]. There is increasing evidence that mast cells promote and maintain gut homeostasis in response to the metabolites and molecules produced by the gut microbiota [6]. Mast cells are located in all tissues, except the blood, and mediate type 2 allergic inflammatory responses by releasing proinflammatory mediators [7]. Mast cells are highly heterogeneous, displaying a variety of phenotypes and functions in these different tissues [8]. Short-chain fatty acids such as butyrate inhibit allergen-induced histamine release and airway contraction in a guinea pig model and inhibit the antigen-dependent and independent activation of mouse and human mast cells [9,10,11,12]. Interestingly, these effects are independent of G protein-coupled receptors (GPR) that bind to short-chain fatty acids (GPR41 and GPR43) and activate functional responses in other cell types [9,13]. Our lab has previously shown that butyrate decreases the proliferation of the human mast cell line (HMC-1) by blocking cell cycle progression [12]. This change in cell proliferation is also associated with phenotypic changes such as increases in tryptase expression and ultrastructural changes in granules, suggesting the maturation of granule contents.

Mast cell granules are packed with glycosaminoglycans (GAGs), which form the matrix within which other mediators, such as histamine and tryptase, are embedded. GAGs are biopolymers composed of long unbranched polysaccharides composed of repeating disaccharide units with viscous properties and usually containing the derivatives of amino sugars and uronic acids. There are four main classes of GAGs: hyaluronan, chondroitin sulfate/dermatan, heparan sulfate/heparin, and keratan sulfate [14]. Hyaluronan is a key mediator in the skin–gut axis, produced during the disruption of the dermis, and causes increased expression in the colon of the host defense genes *Reg3* and *Muc2*, disrupting the gut microbiome [15]. Mucosal mast cell-derived chondroitin sulfate is an important mediator in the expulsion of *Strongyloides venezuelensis* [16]. Heparin is a highly sulfated GAG that is abundant in connective tissue mast cell granules and has the highest negative charge density of any known biological molecule in mammals [17]. Heparin is an anticoagulant and is produced commercially in ton quantities for clinical use [18,19,20].

Although the precise physiologic role of heparin in healthy tissues is still unknown, heparin is an important mediator in the gut, possibly providing an essential communication link between the microbiota and the healthy gut tissue. Heparin binds to flagellated bacteria in the gut [21], and heparin and other GAGs are also essential carbon sources for gut microbiota [22], serving as important mediators of microbiota colonization [23]. It is possible that the gut production of butyrate may facilitate the mast cell release of GAG such as heparin and thereby create an essential regulatory loop to maintain gut health. The relationship between butyrate, mast cells, and the production of GAGs such as heparin is not well understood. Therefore, we hypothesized that butyrate could modify GAG production and storage in human mast cells, possibly by modifying the expression of enzymes associated with GAG biosynthesis.

## 2. Materials and Methods

### 2.1. Cell Culture

#### 2.1.1. HMC-1.2

HMC-1.2 was derived from the peripheral blood of a patient with mast cell leukemia [24] and was a gift from Joseph H. Butterfield (University of Pennsylvania, USA). The HMC-1.2 cells were cultured in Iscove’s Modified Dulbecco’s Medium (IMDM) (Life Technologies, New York, NY, USA) with 5% FBS and 1% penicillin/streptomycin. The HMC-1.2 cells were passaged 2–3 times a week and maintained at a density of 1 × 10^5^ to 1 × 10^6^ cells/mL at 37 °C, 5% CO_2_.

#### 2.1.2. LAD2

LAD2 was cultured as described previously [25]. Briefly, LAD2 was cultured in a serum-free StemPro-34 SFM medium (Thermo Fisher Scientific, Waltham, MA, USA) supplemented with 100 U/mL penicillin, 50 µg/mL streptomycin and 100 ng/mL stem cell factor (SCF) (Peprotech Inc., Rocky Hill, CT, USA), and 2 mM L-glutamine. The cell suspensions were seeded at a density of 1 × 10^5^ cells/mL and maintained at 37 °C and 5% CO_2_. The cells were fed by the hemi-depletion of the medium once per week. The experiments were performed in a StemPro-34 SFM complete medium with 100 ng/mL SCF.

### 2.2. Cholesteryl Butyrate Emulsion Synthesis

Cholesteryl butyrate emulsions (CholButEs) were prepared as described previously [26] with some modifications. Briefly, a warm oil-in-water microemulsion was prepared from cholesteryl butyrate (Sigma-Aldrich, St. Louis, MO, USA, C4758), Epikuron 200 (Cargill, Milan, Italy), sodium taurocholate (Sigma-Aldrich, 86339), and water. Butanol was added as a preservative. The warm microemulsion was dispersed in cold water and the resulting cholesteryl butyrate aqueous dispersion was filtered through fiberglass. CholButEs were washed using a float-a-lyser.

### 2.3. Fluorescence Microscopy

HMC-1.2 (1 × 10^6^ cells/mL) or LAD2 (1 × 10^6^ cells/mL) was treated with 5 µM berberine for 24 h. The cells were washed once with PBS/0.5% BSA and resuspended in PBS/0.5% BSA followed by live cell imaging using the Echo Revolve 4, Hybrid Fluorescence microscope (Discover Echo Inc, 76490-302, San Diego, CA, USA) at 20×. Images in the same field of view were acquired using either transilluminated light (Brightfeild) or 516 nm to visualize the berberine’s fluorescence.

### 2.4. Trypan Blue Exclusion Assay for Cell Viability and Proliferation

HMC-1.2 was seeded at a density of 1 × 10^5^ cells/mL and incubated with 1 mM NaBu (NaBu, Sigma-Aldrich) for 24, 48, or 72 h. Cell counts were performed at 24, 48, and 72 h post treatment using a hemocytometer with trypan blue dye. For experiments involving CholButE, the HMC-1.2 cells were seeded at a density of 1  × 10^5^ cells/200 µL and treated with varying concentrations of CholButE (24 µM and 120 µM) for 24, 48, and 72 h. After 48 h sample collection, the cells were centrifuged at 200× *g* for 5 min at RT followed by replacing 150 µL old media with fresh media. Cell counts were performed at 24, 48, and 72 h post treatment using a hemocytometer and trypan blue dye to stain the dead cells.

### 2.5. XTT Assay for Cell Metabolic Activity

HMC-1.2 was seeded at a density of 1 ×  10^5^ cells/mL and incubated with 1 mM NaBu for 24, 48, or 72 h. One hundred µL cells were collected and added to 50 µL of XTT reagent (Roche, Laval, QC, Canada) in a 96-well plate and incubated for 4 h at 37 °C and 5% CO_2_. For CholButE, the HMC-1.2 cells were seeded at a density of 1  × 10^5^ cells/200 µL well in a 96-well plate and treated with 24 µM and 120 µM CholButE for 24 or 48 h. At 24 or 48 h, the cells were centrifuged at 200× *g* for 5 min at RT, 100 µL was removed, and 50 µL of XTT reagent (Roche, Laval, QC, Canada) was added. The cells and XTT reagent were incubated for 4 h at 37 °C and 5% CO_2_. Absorbance was measured at 405 nm using a VarioSkan Lux plate reader (ThermoFisher, Waltham, MA, USA) and the results were plotted using the Microsoft Excel software (Microsoft Office LTSC Professional Plus 2021).

### 2.6. Flow Cytometry

HMC-1.2 cells (1 × 10^6^ cells/mL) were incubated with 0, 0.01, 0.1, and 1 mM NaBu or 0, 2.4, 24, and 120 µM CholButE for 48 h followed by treatment with 5 µM berberine for 24 h unless otherwise stated. LAD2 (100,000) cells were incubated with 0, 0.1, 0.5, and 1 mM NaBu or 0, 2.4, 24, and 120 µM CholButE for 48 h followed by treatment with 5 µM berberine for 24 h. The cells were washed twice with PBS/0.5% BSA, resuspended in PBS/0.5% BSA, followed by flow cytometry using the Cytoflex flow cytometer (Beckman Coulter, Indianapolis, IN, USA) equipped with an Argon ion laser (488–514 nm) and bandpass filter to enable detection fluorescence emission at 516 nm (for GFP) and 775 nm (for near-IR dead cell stain). The fluorescence of 20,000 cells was acquired at a medium flow rate using the Cytoflex flow cytometer and data analysis was performed using the FlowJo software version 10.6.2 (Becton, Dickinson and Company, Ashland, OR, USA). The cells and debris were gated on an SSC-A vs. FSC-A plot and removed from the final analysis. For immunolabeling, 100,000 HMC-1.2 cells in PBS/0.5% BSA were labeled with a 1:1 mixture of anti-hu FcεRIα-APC (eBioScience, Cartsbad, CA, USA, 17-5899-42) and anti-hu CD117 (c-Kit)-PE (eBioScience, 12-1179-41) antibodies or mouse IgG2b κ APC (eBioScience, 17-4732-42) and mouse IgG1 κ isotype PE (eBioScience, 12-4714-81) isotype controls for 1 h at 4 °C in the dark. After immunolabeling, the HMC-1.2 cells were washed 3X with PBS/0.5% BSA and analyzed on a flow cytometer as described above. For staining purposes, 100,000 HMC-1.2 cells were stained with the indicated concentrations of Calcein acetoxymethyl (Invitrogen, Eugene, OR, USA, C3100MP), Nile red (Sigma-Aldrich, 72485), and propidium iodide (Invitrogen, P3566) followed by flow cytometry.

### 2.7. Near-IR Dead Cell Staining to Determine Cell Membrane Integrity

HMC-1.2 (100,000/100 µL) was incubated with 0.1 µL of a freshly reconstituted Near-IR dead cell stain (Invitrogen, L34975) for 30 min at room temperature (RT). The cells were washed twice in PBS/BSA and the fluorescence of 20,000 cells was measured using the Cytoflex flow cytometer as described above. The data were analyzed using FlowJo 10.6.2 as described above.

### 2.8. RNA Extraction, cDNA Synthesis and qRT-PCR

Four million cells were centrifuged at 5000× *g* for 5 min, the supernatant was removed, and the cells were lysed in an RLT buffer (Qiagen, Hilden, Germany) containing β-mercaptoethanol. RNA was extracted using the RNeasy Mini Kit (Qiagen, Hilden, Germany) that employed on-column DNase (Qiagen, Hilden, Germany) digestion. The purity and concentration of the RNA were determined using Nanodrop One (ThermoFisher) and cDNA was synthesized using 1000 ng of total RNA by utilizing the High-capacity cDNA reverse transcription kit (Applied Biosystems, Waltham, MA, USA). qRT-PCR was performed with the Fast SYBR Green master mix (Applied Biosystems), 20 ng of cDNA, gene-specific IDT oligonucleotide primers as described in Table 1, and a StepOnePlus real-time PCR machine (Applied Biosystems). The GAPDH primers as reported previously were utilized as an internal control to normalize the samples [25]. The data were analyzed by using the 2^−ΔΔCT^ method.

For the analysis of HMC-1.2 and LAD2 *Tryptase alpha/beta 1*, *TPSAB1 (Tryptase)*, *Chymase 1*, *CMA1 (Chymase)*, and *Mas-related G protein-coupled receptor* (*MRGPRX2*) mRNA expression, total RNA was extracted from two independent HMC-1.2 or LAD2 cultures. cDNA was synthesized from 1000 ng total RNA. qRT-PCR was performed using 20 ng of cDNA, Prime time gene expression assay kit (IDT, 1055772), and Taqman probes specific to human *TPSAB1*, *CMA1*, *MRGPRX2*, *β-actin* [27,28], and *GapDH* (Applied Biosystems, P/N 4310859, Batch 0004004). Fold change in the *TPSAB1* and *MRGPRX2* expressions was analyzed by using the 2^−ΔΔCT^ method relative to the *CMA1*.

### 2.9. ^1^H NMR Spectroscopic Study of Intermolecular Interaction between Berberine and Heparin

An NMR solution of 50% methanol-d4 and 50% D_2_O containing 3-(trimethylsilyl)-propionic-d_4_ (TMSP-d4, Sigma-Aldrich) acid sodium salt (0.087 mM) as an internal standard was prepared. A 10.0 mM berberine chloride (Sigma-Aldrich) stock solution was prepared using the NMR solution, and a 1.0 mM berberine chloride solution was utilized for the berberine NMR measurement. Similarly, a stock solution of 10.0 mg/mL heparin (Sigma-Aldrich, H4784) was prepared in the NMR solution, and a 5.0 mg/mL solution was used for the heparin NMR measurement. A sample containing 1.0 mM berberine and 5.0 mg/mL heparin was prepared from the corresponding stock solutions.

All the NMR spectra were recorded on a Varian Direct Drive VNMRS 600 spectrometer operating at a magnetic field strength of 14.1 T (599.49 MHz proton frequency) and equipped with an autoX dual broadband probe. One dimensional (1D) ^1^H NMR spectra were measured for all the samples using multiple frequency solvent suppression (Presat) at 298 K. TMSP-d_4_ was used as the internal reference standard (0 ppm) for spectra calibration.

### 2.10. HPLC Analysis of Heparin in the Cell Lysate of HMC-1.2

Heparin sodium salt from porcine intestinal mucosa (>180 USP units/mg), sodium phosphate dibasic heptahydrate, NaCl, and sodium hydroxide were purchased from Sigma-Aldrich. Separations were conducted on an Agilent 1260 HPLC system. An Agilent Zorbax NH_2_ column (4.6 *×* 250 mm, 5.0 µm) was used for the heparin separation. The mobile phase was 0.1 M NaCl in a 0.1 mM HPO_4_^2−^ buffer with a pH of 9.65 at a flow rate of 0.5 mL/min. The volume of injection was 20 µL. Analyte detection was achieved using UV absorption at 215 nm. Identification and quantification were achieved by the comparison of peak retention time, and the area of reference standards.

HMC-1.2 cells were harvested via centrifugation at 200× *g* for 5 min after the 48 or 72 h treatment with PBS or 1.0 mM NaBu. The supernatant was discarded, and the cells were washed 3× with 10 mL of PBS. Following another centrifugation at 200× *g* for 5 min, the cells were stored at −80 °C until analysis. The cells were lysed via three cycles of freeze–thaw procedure. The cells were then resuspended in a PBS buffer. The mixture was vigorously mixed and vortexed for 1 min at the highest setting. Cell debris was removed by ultracentrifugation at 16,000× *g* for 15 min at 4 °C. The supernatant was filtered by a 0.45 µm PTFE 13 mm membrane and collected in a 2.0 mL vial for HPLC analysis. A cell density equivalent to 13.7 × 10^6^ cells/mL was utilized to obtain a chromatogram from untreated (Ut) or NaBu-treated HMC-1.2 lysates for comparison. The absolute concentration of heparin (in mg/mL/10 × 10^6^ cells/mL) was obtained by using the linear fit equation “y = 398.53898X + 3.2369” based on the standard calibration curve generated using 0.1–2 mg/mL heparin. For data analysis, the absolute heparin levels obtained in 1 mM NaBu-treated 48 h and 72 h samples were divided with those of the corresponding Ut samples to obtain fold change in heparin content.

### 2.11. Statistical Analysis

The experiments were conducted from at least three independent cultures of cells and the values represent the mean of n = 3–7 + the standard error of the mean. *p* values were determined by one-way ANOVA (between groups) or Student’s *t*-test using the GraphPad prism software (https://www.graphpad.com/quickcalcs/ttest1/?format=C).

## 3. Results

### 3.1. Characterization of HMC-1.2

**Figure 1:**
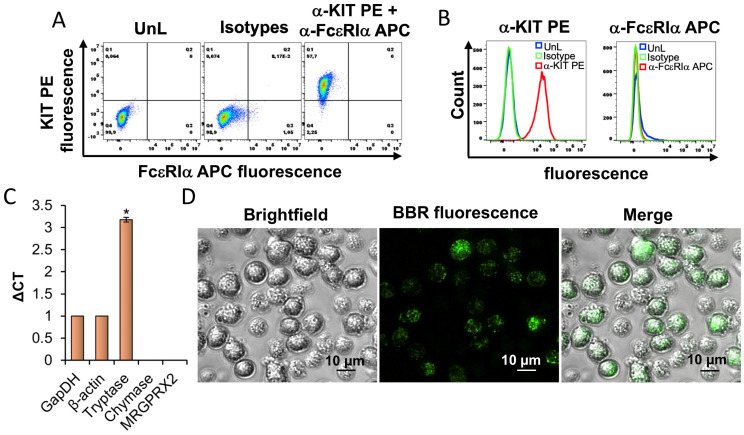
Characterization of HMC-1.2. (**A**) In total, 100,000 HMC-1.2 cells in a PBS/BSA buffer were immunolabeled with a-KIT PE+ a-FcεRIα APC antibodies or the corresponding isotype controls for 1 h at 4 °C in the dark. The HMC-1.2 cells were washed 3× with the PBS/BSA buffer and processed for flow cytometry. UnL represents unlabeled cells. A total of 20,000 cells were analyzed to determine (**A**) KIT PE versus FcεRIα APC fluorescence dot plots or (**B**) histogram overlay showing KIT PE or FcεRIα APC fluorescence of the UnL isotype or a-KIT PE+ a-FcεRIα APC immunolabeled HMC-1.2. The data shown in “A” and “B” are representative of three independent experiments. (**C**) The qRT-PCR analysis of human *TPSAB1 (Tryptase)*, *CMA1 (Chymase)*, and *MRGPRX2* mRNA expression in HMC-1.2. *GapDH* and *β-actin* were utilized as endogenous controls to normalize the samples. The data from two independent cultures is represented as average delta CT (critical threshold). A Student’s *t*-test was conducted to calculate statistical significance (*p* < 0.05 = *) relative to *GapDH* and *β-actin*. Note: HMC-1.2 does not express *CMA1 (Chymase)* and *MRGPRX2*. (**D**) A total of 100,000 HMC-1.2 cells were stained with 5 µM BBR for 24 h and analyzed under the Echo Revolve 4 hybrid fluorescence microscope at 20× magnification. The scale represents 10 µm.

HMC-1.2 cells were analyzed for their expression of the quintessential mast cell surface receptors FcεRI and KIT by flow cytometry. Results show that ~97.7% of HMC-1.2 express KIT receptors on their surface as indicated by the dot plots and histograms (Figure 1A,B). However, HMC-1.2 does not appear to express the FcεRI receptor (Figure 1A,B) as shown previously [29]. Furthermore, we analyzed *Tryptase alpha/beta 1*, *TPSAB1 (Tryptase)*, *Chymase 1*, *CMA1 (Chymase)*, and *Mas-related G protein-coupled receptor* (*MRGPRX2*) mRNA expression in the HMC-1.2 cultures using a qRT-PCR analysis. Our results show that HMC-1.2 expresses 3-fold higher *tryptase* mRNA relative to housekeeping genes *GapDH* or *β-actin* (Figure 1C). However, *chymase* and *MRGPRX2* were not detected in our analysis confirming previous observations [29]. To visualize GAGs, we utilized berberine (BBR), a naturally occurring fluorescent cationic benzylisoquinoline compound that has been shown to bind sulfated GAGs such as heparin and heparan sulfate present inside granules [30,31]. HMC-1.2 cells were stained with berberine for 24 h followed by live cell imaging using fluorescence microscopy. Figure 1D shows that the berberine interacts with the sulfated GAGs within the HMC-1.2 granules as indicated by punctate green dots (Figure 1D). These results reinforce previous observations that berberine interacts with heparin within mast cell granules [30,31].

### 3.2. Berberine Interacts with Heparin

**Figure 2:**
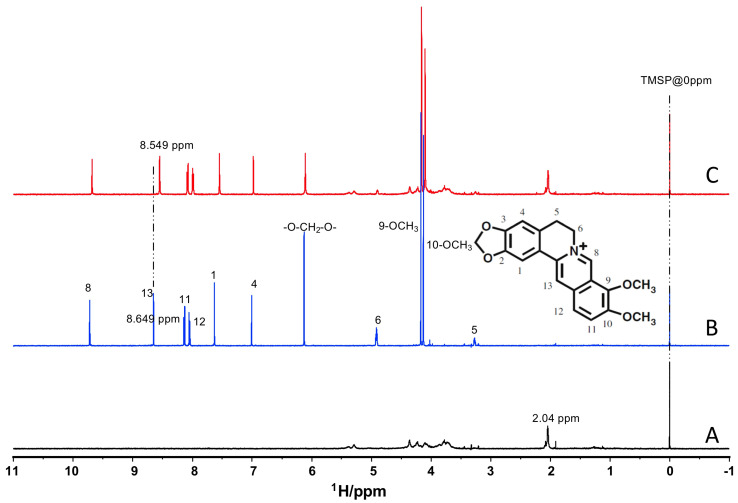
Berberine interacts with heparin in vitro. The ^1^H NMR spectra of 5.0 mg/mL heparin (**A**), 1.0 mM berberine (**B**), and (**C**) the mixture solution containing 1.0 mM berberine and 5.0 mg/mL heparin. The chemical structure of berberine is shown as an inset in (**B**).

To evaluate if berberine interacts with heparin, NMR studies were conducted using heparin, berberine, or a 1:1 mixture of heparin and berberine. A typical ^1^H NMR spectrum was observed for heparin with broad lines due to its polymeric nature (Figure 2A). The strong peak at 2.04 ppm corresponds to the methyl signal of N-acetylated glucosamine (COCH_3_) [32]. Various signals obtained in the ^1^H NMR spectrum of berberine (Figure 2B) were assigned and labeled [33]. In the presence of heparin, the ^1^H NMR spectrum of all the berberine signals were shifted to a higher field (Figure 2C). The maximum chemical shift change was observed with the H13 (8.549 ppm) and H1 (7.628 ppm) signals (Table 2). These results suggest a strong intermolecular interaction between berberine and heparin since the presence of heparin would have a significant shielding effect on the berberine molecules, particularly in the positions H13, H1, H11, and H12.

### 3.3. NaBu Treatment Increases Berberine Fluorescence and Granularity of HMC-1.2

**Figure 3:**
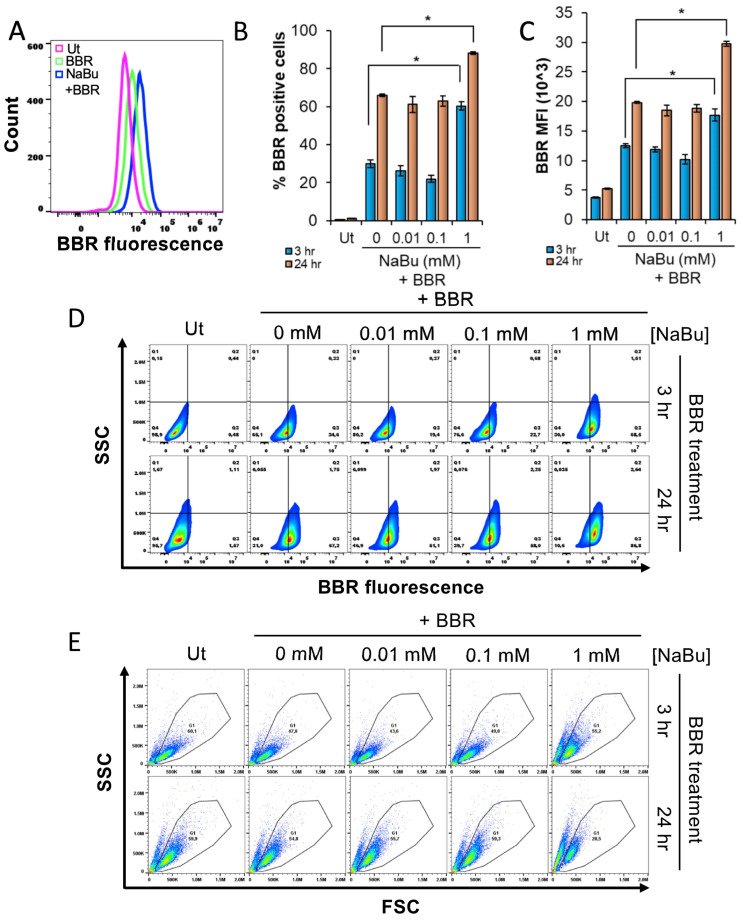
Sodium butyrate (NaBu) enhances berberine (BBR) fluorescence in HMC-1.2 and increases granularity (**A**) A total of 100,000 HMC-1.2 cells were incubated with 1 mM NaBu for 48 h followed by treatment with 5 µM BBR for 24 h. The cells were collected and processed for flow cytometry to visualize the BBR fluorescence histogram. (**B**–**E**) A total of 100,000 HMC-1.2 cells were incubated with 0, 0.01, 0.1, or 1 mM NaBu for 48 h followed by treatment with 5 µM BBR for 3 h or 24 h. The cells were collected and processed for flow cytometry. In total, 20,000 cells were analyzed to determine (**B**) % BBR positive cells, (**C**) BBR mean fluorescence intensity (MFI), (**D**) side scatter (SSC) versus BBR fluorescence contour plots, and (**E**) forward scatter (FSC) vs. side scatter (SSC) dot plots. Ut represents “untreated cells”. n = 3; a Student’s *t*-test was performed to determine the statistical significance (*p* < 0.05 = *) relative to the 0 mM NaBu+BBR samples of corresponding time points in B and C. The data shown in (**A**,**D**,**E**) are representative of three independent experiments.

HMC-1.2 cells were incubated with 1 mM NaBu for 24 h followed by treatment with 5 µM berberine for 24 h. The flow cytometric analysis showed that HMC-1.2 appeared viable (Figure S1A) at 1 mM NaBu treatment and that there was no effect of NaBu in berberine fluorescence (Figure S1A). However, when the time of the NaBu treatment was increased up to 48 h, a significant proportion of HMC-1.2 showed increased berberine fluorescence as indicated by a shift of the berberine fluorescence histogram towards the right (Figure 3A), suggesting that the NaBu-mediated increase in berberine fluorescence is a time-dependent process that requires at least 24–48 h for optimal effect. The cell viability (Figure S1C) and metabolic activity (Figure S1D) analysis showed that the cells were viable and metabolically active up to 72 and 48 h, respectively, post 1 mM NaBu treatment.

To further evaluate the effects of NaBu, HMC-1.2 cells were treated with varying concentrations of NaBu (0.01, 0.1, or 1 mM) for 48 h followed by measuring berberine fluorescence at 3 or 24 h post berberine treatment. Figure 3B,C show that at 0.1 and 1 mM NaBu, the percent of berberine positive cells as well as berberine mean fluorescence intensity (MFI), respectively, increased in a time-dependent manner; contour plot analysis (Figure 3D) reinforced this observation. However, an increase in berberine fluorescence was not observed at the 0.01 mM or 100 µM NaBu treatment, suggesting that the NaBu-mediated increase in berberine fluorescence required a minimum threshold concentration. Furthermore, side scatter (indicative of cell complexity/granularity) versus forward scatter (FSC, indicative of cell size) dot plot analysis indicated that the 1 mM NaBu treatment increased the complexity/granularity of the cells as evidenced by a shift of the cells towards the left in the gated population at both 3 and 24 h post berberine treatment (Figure 3E). This prominent change in cell granularity was not observed with the 24 h NaBu treatment (Figure S1A). Collectively, these results suggest that a NaBu-mediated increase in berberine fluorescence corresponds to an increase in cell granularity.

### 3.4. NaBu Increases Heparin Content of HMC-1.2

**Figure 4:**
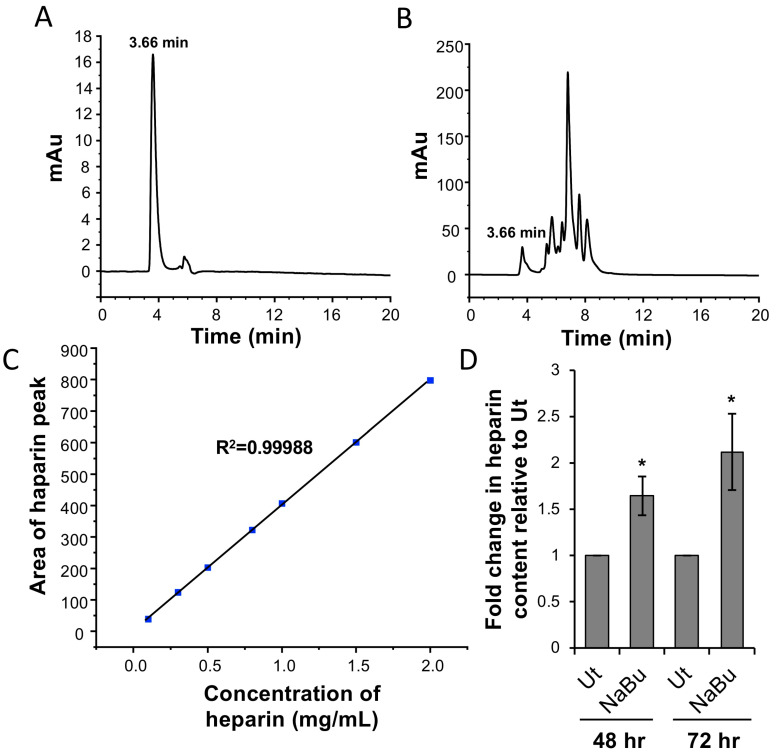
NaBu increases the heparin content of HMC-1.2. A representative UV-detected chromatogram of heparin ((**A**), 1 mg/mL) and HMC-1.2 lysate (**B**). (**C**) The standard calibration curve utilized for evaluating the absolute concentration of heparin in the HMC-1.2 lysates using HPLC analysis. (**D**) The fold change in the heparin content after 1 mM NaBu treatment for 48 h and 72 h relative to untreated (Ut) (**D**). A Student’s *t*-test was conducted to evaluate statistical significance. n = 5, *p* < 0.05 = *.

To evaluate if the NaBu-mediated increase in berberine fluorescence and granularity is associated with an increase in heparin content in HMC-1.2, an HPLC analysis was conducted. HMC-1.2 cells were treated with 1 mM NaBu or PBS (untreated, Ut) for 48 or 72 h followed by HPLC analysis using cell lysates. For analysis purposes, a UV-detected chromatogram of 1 mg/mL heparin standard was evaluated (Figure 4A). A retention time of 3.66 min was recorded for the heparin peak. For the HMC-1.2 lysates, a peak at 3.66 min retention time corresponding to heparin was observed including several other peaks (Figure 4B). To quantify the amount of heparin present in the HMC-1.2 lysates, a standard calibration curve was generated with 0.1–2.0 mg/mL heparin (Figure 4C). The level of heparin in HMC-1.2 significantly increased by 1.6 ± 0.2-fold at 48 h and 2.11 ± 0.4-fold at 72 h relative to untreated (Figure 4D). Moreover, a greater increase was observed at 72 h as compared to 48 h. In summary, these results suggest that NaBu treatment increases the heparin content of HMC-1.2.

### 3.5. NaBu-Treated HMC-1.2 Have Intact Cell Membrane

**Figure 5:**
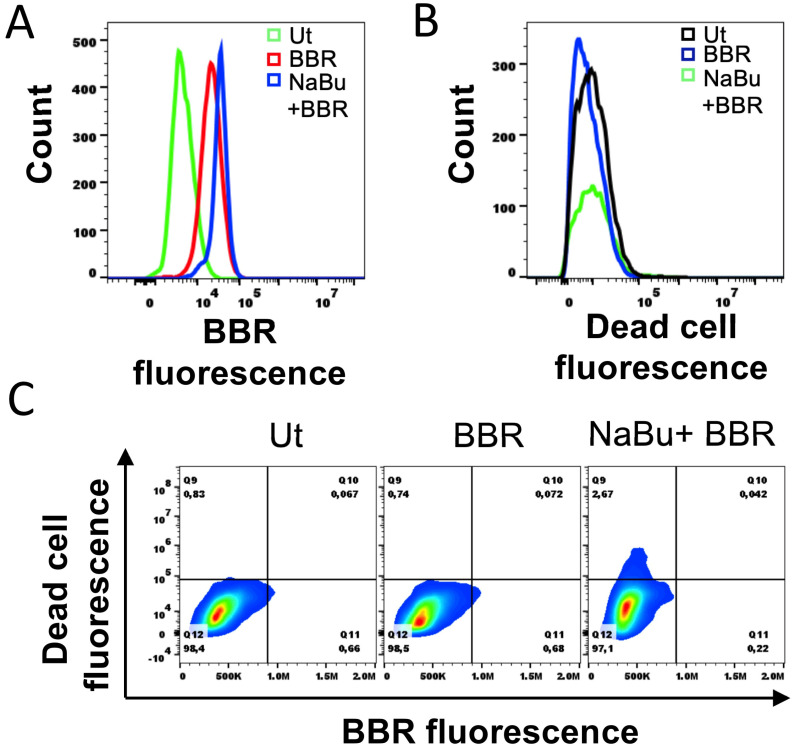
The 1 mM NaBu-treated HMC-1.2 cells have intact cell membranes. (**A**) A total of 100,000 HMC-1.2 cells were treated with 1 mM NaBu for 48 h followed by treatment with 5 µM BBR for 24 h. The cells were collected, washed twice with PBS/BSA, and stained with free amine-reactive near-IR dead cell stain for 30 min at RT in the dark. After staining, the cells were washed twice with PBS/BSA and processed for flow cytometry; 20,000 cells were analyzed for (**A**) BBR fluorescence, (**B**) dead cell fluorescence, and (**C**) dead cell versus BBR fluorescence. Ut represents “untreated cells”. The data shown is representative of three independent experiments.

Next, we hypothesized that the NaBu-mediated increase in berberine fluorescence could result from the increased internalization of berberine in HMC-1.2 with compromised cell membranes. To test this possibility, we utilized a free amine-reactive near-IR dead cell fluorescent dye that binds significantly more free amines in cells with compromised membranes as compared to healthy cells with intact cell membranes. HMC-1.2 cells were incubated with 0.1 or 1 mM HMC-1.2 for 48 h prior to treatment with 5 µM berberine for 24 h and staining with the free amine dye to simultaneously visualize the berberine (Figure 5A) and dead cell (Figure 5B) fluorescence histograms. The histogram overlay results show that unlike berberine (Figure 5A), dead cell fluorescence did not change upon the 1 mM NaBu treatment (Figure 5B), suggesting that the observed increase in berberine staining is likely due to an increase in the granular content in HMC-1.2 rather than a compromised cell membrane. The dead cell versus berberine fluorescence contour plots (Figure 5C) further showed that the majority of berberine fluorescence was emitted by healthy cells with an intact cell membrane (Quadrant Q7, indicated by the orange, yellow, and green sub-populations in the contour plot).

### 3.6. NaBu Increases the Production of Genes Involved in Sulfated GAG Biosynthesis

**Figure 6:**
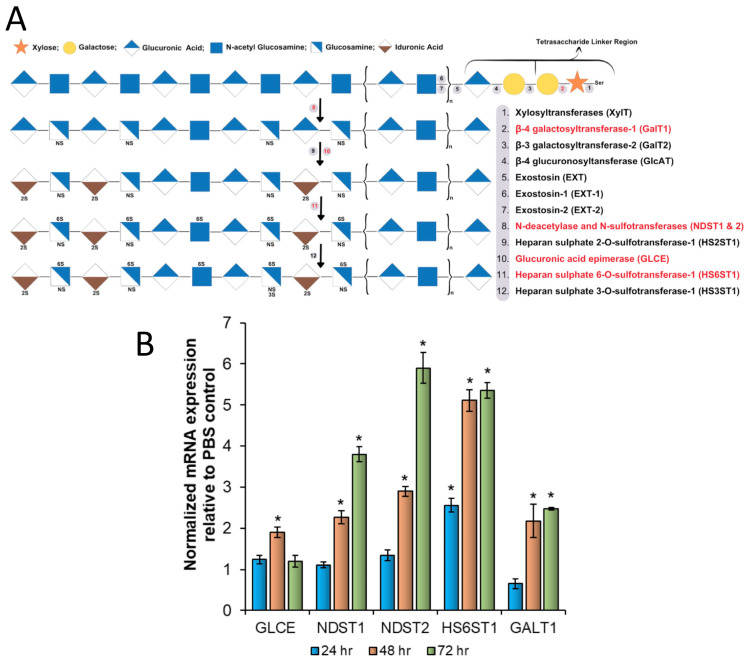
NaBu increases the mRNA expression of genes involved in sulfated GAG (heparin and heparan sulfate) biosynthesis. (**A**) The metabolic pathway involved in the biosynthesis pathway of highly sulfated GAGs such as heparin and heparan sulfate. The 2S, 3S, and 6S, respectively, refer to 2-O, 3-O, and 6-O-sulfation, whilst NS refers to N-sulfated glucosamine. The enzymes that were included in our analysis are highlighted in red. (**B**) A total of 4 million HMC-1.2 cells were treated with 1 mM NaBu for 24, 48, or 72 h followed by total RNA extraction, cDNA synthesis using 1000 ng total RNA, and qRT-PCR using gene-specific primers. *GapDH* was used as an internal control to normalize the samples. The data were analyzed using the 2^−ΔΔCT^ method. n = 4. A Student’s *t*-test was conducted to calculate the statistical significance (*p* < 0.05 = *) relative to the mRNA expression in the PBS control of the corresponding time point. A similar trend in mRNA expression was obtained in an independent biological replicate.

The results shown thus far suggested that the NaBu-mediated increase in berberine fluorescence was associated with an increase in HMC-1.2 granularity and heparin content. Hence, we hypothesized that NaBu treatment increased the synthesis of the genes involved in sulfated GAGs such as heparin and heparan sulfate that are present inside granules. Heparin and heparan sulfate are biosynthesized in the endoplasmic reticulum and Golgi by a common pathway in a highly complex and dynamic process that involves over 20 different enzymes [34,35,36,37,38]. The metabolic pathway for the biosynthesis of heparin and heparan sulfate involves the coordinated action of several key enzymes as shown in Figure 6A. To evaluate the effects of NaBu on the mRNA expression of enzymes involved in the biosynthesis of heparin/heparan sulfate, HMC-1.2 cells were treated with 1 mM NaBu for 24, 48, and 72 h followed by RNA extraction, cDNA synthesis, and qRT-PCR to evaluate the gene expression of glucuronic acid epimerase (*GLCE*), N-deacetylase and N-sulfotransferase 1 (*NDST1*), N-deacetylase and N-sulfotransferase 2 (*NDST2*), heparan sulfate 6-O-sulfotransferase 1 (*HS6ST1*), and galactosyltransferase I (*GALT1*). Glyceraldehyde 3-phosphate dehydrogenase (*GAPDH*) was utilized as an internal control. Figure 6B shows that the mRNA expression of *GLCE*, *NDST1*, *NDST2*, *HS6ST1*, and *GALT1* changed differentially upon the NaBu treatment. At 24 h post NaBu treatment, the mRNA expression of most of the tested genes was unaltered except *HS6ST1* which was induced up to 2.5-fold. By 48 h post NaBu treatment, the expression of all the tested genes was significantly upregulated by 2- to 5-fold compared to the untreated cells, with *HS6ST1* still at the highest expression. By 72 h, there was a time-dependent increase in the expression pattern of all the genes with the exception of *GLCE* which was reduced at 72 h as compared to 48 h. At 72 h, *NDST2* was induced to its maximum expression by approximately 6-fold compared to the untreated cells.

### 3.7. Cholesteryl Butyrate Emulsions (CholButE) Increase Berberine Fluorescence

**Figure 7:**
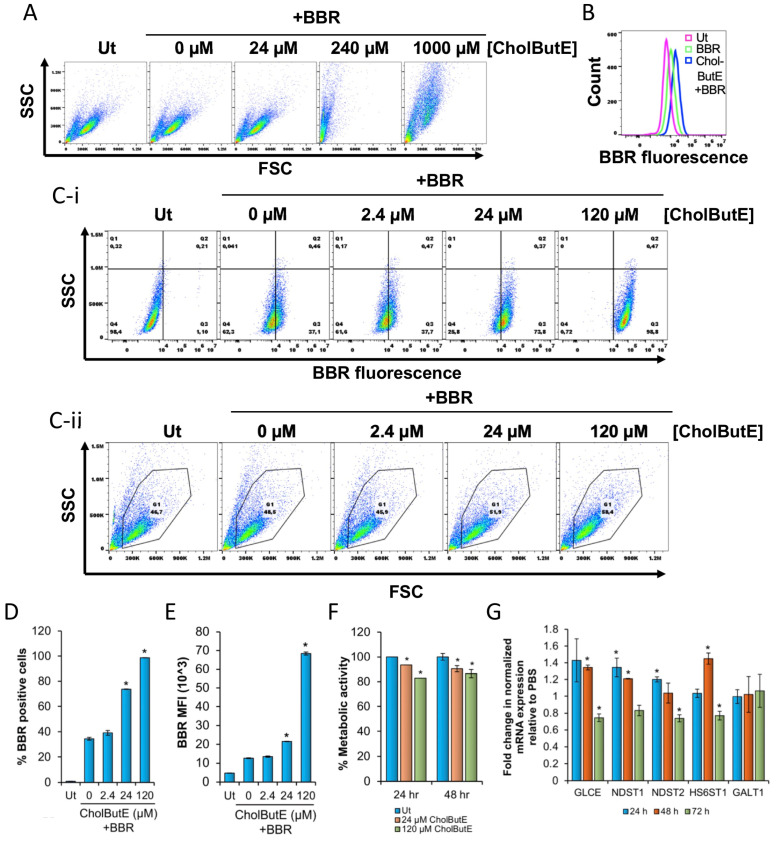
Cholesteryl butyrate emulsions (CholButE) increase BBR fluorescence. (**A**) A total of 100,000 HMC-1.2 cells were treated with 0, 24, 240, or 1000 µM CholButE for 48 h followed by treatment with 1 µM BBR for 24 h. In total, 20,000 cells were analyzed to determine SSC vs. FSC. (**B**) The histogram overlay of untreated (Ut), BBR-, or 24 µM CholButE+ BBR-treated HMC-1.2 as in A. (**C**–**E**) A total of 100,000 HMC-1.2 cells were treated with 0, 2.4, 24, or 120 µM CholButE for 48 h followed by treatment with 5 µM BBR for 24 h and processed for flow cytometry to determine (**C-i**) SSC vs. BBR fluorescence dot plots, (**C-ii**) SSC vs. FSC dot plot, (**D**) % BBR positive cells, or (**E**) BBR MFI. (**F**) A total of 100,000 HMC-1.2 cells were treated with 24 or 120 µM CholButE for 24 or 48 h followed by measuring metabolic activity using the XTT assay. (**G**) A total of 4 million HMC-1.2 cells were treated with 120 µM CholButE for 24, 48, or 72 h followed by total RNA extraction, cDNA synthesis, and qRT-PCR using gene-specific primers. *GapDH* was used as an internal control to normalize the samples. Data were analyzed using the 2^−ΔΔCT^ method. n = 3–4. A Student’s *t*-test was conducted to calculate the statistical significance (*p* < 0.05 = *) relative to the 0 µM CholButE + BBR samples in (**D**,**E**), Ut samples in (**F**), or mRNA expression observed in PBS control of corresponding time point. A similar trend in mRNA expression was obtained in an independent biological replicate. The data shown in (**A**,**C**) is representative of three independent experiments.

CholButE was synthesized using a protocol modified from Minellie et al. [26]. HMC-1.2 cells were treated with varying concentrations of CholButE (24, 240, and 1000 µM) for 48 h followed by berberine treatment. The flow cytometric analysis showed that 240 µM and 1000 µM CholButE were toxic to HMC-1.2 as indicated by a significant decrease in the SSC and FSC, which indicates a decrease in cell size and intracellular granularity and a decrease in viability (Figure 7A). However, after the 24 µM CholButE treatment, HMC-1.2 appeared viable and showed an increase in berberine fluorescence as compared to the CholButE-untreated cells (Figure 7B).

To examine the effects of CholButE on berberine fluorescence, HMC-1.2 cells were treated with 120, 24, and 2.4 µM CholButE for 48 h followed by treatment with berberine for 24 h. The flow cytometric analysis showed that there was a concentration-dependent significant increase in the percent of cells that internalized berberine (Figure 7C,D) as well as berberine fluorescence (Figure 7E) with the most prominent effects seen at 120 µM CholButE. However, unlike NaBu, the CholButE treatment did not cause a significant change in SSC (Figure 7C-ii), suggesting that HMC-1.2 granularity was modestly changed. Furthermore, a similar effect in berberine fluorescence was observed at 24 h post CholButE treatment (Figure S2C), suggesting that CholButE increased berberine fluorescence in a concentration-dependent manner at both 24 and 48 h post incubation.

To evaluate the effects of CholButE on metabolic activity, HMC-1.2 cells were treated with 24 or 120 µM CholButE for 24 and 48 h followed by the analysis of metabolic activity by an XTT assay. Figure 7F shows that there is a significant reduction in metabolic activity after 24 and 48 h treatment with CholButE. Even though metabolic activity remained greater than 80% at all times, the most significant reduction in metabolic activity occurred when the cells were treated with 120 µM CholButE. These results suggest that CholButE alters the metabolic activity of HMC-1.2.

Additionally, 120 µM CholButE modified the expression of the enzymes associated with GAG synthesis (Figure 7G). At 24 h post CholButE treatment, the mRNA expressions of *NDST1* and *NDST2* were increased relative to the PBS control. By 48 h post CholButE treatment, the mRNA expression of *GLCE* and *HS6ST1* were increased. The expression of *GLCE*, *NDST1*, and *HS6ST1* mRNA was reduced by 72 h. The expression level of *GALT1* was unaltered at all of the time points.

### 3.8. Cholesteryl Butyrate Emulsion Does Not Decrease Cell Viability or Cause Internalization of Lipophilic Dyes

**Figure 8:**
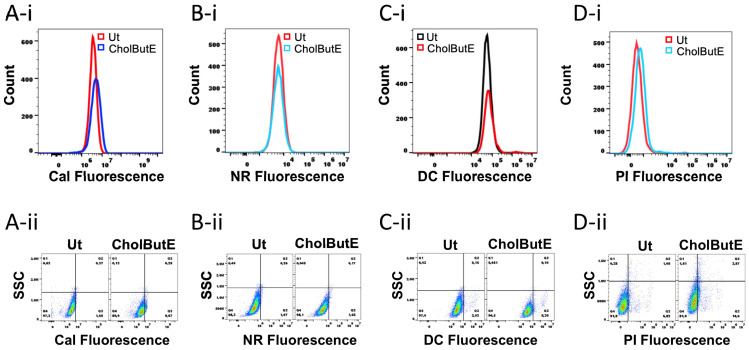
CholButE-mediated increase in fluorescence is specific to BBR: (**A**) A total of 100,000 HMC-1.2 cells were treated with 120 µM CholButE for 24 h. The cells were collected and washed 2× with PBS/BSA prior to staining with fluorochromes. The untreated (Ut) or CholButE-treated cells were stained with (**A**) 5 µM calcein acetoxymethyl (Cal) for 30 min at RT, (**B**) 1 µM Nile red (NR) for 30 min at RT, (**C**) 0.1 µL of a freshly reconstituted free amine-reactive near-IR dead cell (DC) stain for 30 min, and (**D**) 10 µg/mL propidium iodide (PI) for 1 h at 4 °C in the dark. The DC- and NR-stained cells were washed twice before data acquisition. The PI- and Cal-stained cells were analyzed directly on the flow cytometer. In total, 20,000 cells were acquired to visualize the histograms (**A-i**,**B-i**,**C-i**,**D-i**) and dot plots (**A-ii**,**B-ii**,**C-ii**,**D-ii**) of respective fluorochromes. Each dataset was analyzed relative to the untreated HMC-1.2 (Ut) cells that were stained with the corresponding fluorochromes. The data presented is representative of three independent experiments.

Next, we determined whether the CholButE-mediated increase in berberine fluorescence was specific, or whether CholButE would increase the internalization of other dyes that did not bind to GAGs. HMC-1.2 cells were treated with 120 µM CholButE for 24 h followed by staining with different fluorochromes such as propidium iodide (PI), calcein acetoxymethyl (Cal), Nile red (NR), or free amine-binding near-IR dead cell (DC) stain. Untreated HMC-1.2 (Ut) cells were stained with the corresponding fluorochromes for data analysis. The CholButE-treated HMC-1.2 cells were also treated with 50 µM berberine for 3 h. As expected, the CholButE treatment showed a significant increase in berberine fluorescence (Figure S3). For the data analysis of other fluorochromes, the untreated HMC-1.2 cells were arbitrarily assigned approximately 1–7% fluorescent cells (Figure 8A–D, Q3) to visualize changes in the number of fluorescent cells in CholButE-treated HMC-1.2. The histogram overlay and dot plot analysis show that CholButE did not significantly increase the internalization of the passively diffusing dye calcein acetoxymethyl (Cal, Figure 8A). CholButE did not increase the binding of the lipophilic dye Nile red (NR, Figure 8B), or free amine-binding near-IR dead cell stain (DC, Figure 8C). Finally, CholButE did not significantly increase the internalization of lipid-impermeant propidium iodide (PI, Figure 8D), suggesting that the cell membrane remained intact.

### 3.9. Cholesteryl Butyrate Emulsion Reduces Proliferation of HMC-1.2 without Reducing Cell Viability or Metabolic Activity

**Figure 9:**
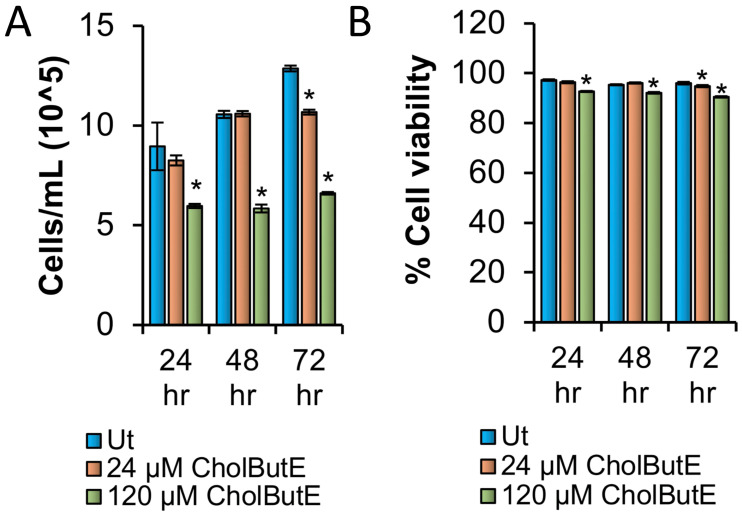
CholButE reduces HMC-1.2 proliferation without affecting viability. (**A**,**B**) A total of 100,000 HMC-1.2 cells were treated with 24 or 120 µM CholButE for 24, 48, or 72 h followed by measuring cell proliferation (**A**) or viability (**B**) using trypan blue exclusion assay. Ut represents “untreated cells”. n = 4; a Student’s *t*-test was performed to determine the statistical significance (*p* < 0.05 = *) relative to the Ut samples of the corresponding time points.

Previous work in our lab shows that NaBu can reduce the proliferation of HMC-1.2 [12]. To evaluate if CholButE has a similar effect, HMC-1.2 cells were treated with 24 or 120 µM CholButE for 24, 48, or 72 h followed by measuring cell proliferation using trypan blue stain. Figure 9A shows that 120 µM CholButE significantly reduced HMC-1.2 proliferation at 24, 48, and 72 h. With the 24 µM CholButE concentration, no effects were observed up to 48 h. However, a significant reduction in cell proliferation was observed at 72 h post treatment. Moreover, 24 and 120 µM CholButE had minimal effects (>90% at all the time points at 24 and 120 µM CholButE concentrations) on cell viability at all the tested time points (Figure 9B), suggesting that CholButE reduces HMC-1.2 proliferation without substantially affecting cell viability.

### 3.10. Sodium Butyrate (NaBu) and Cholesteryl Butyrate Emulsion (CholButE) Modestly Increase BBR Fluorescence in LAD2

**Figure 10:**
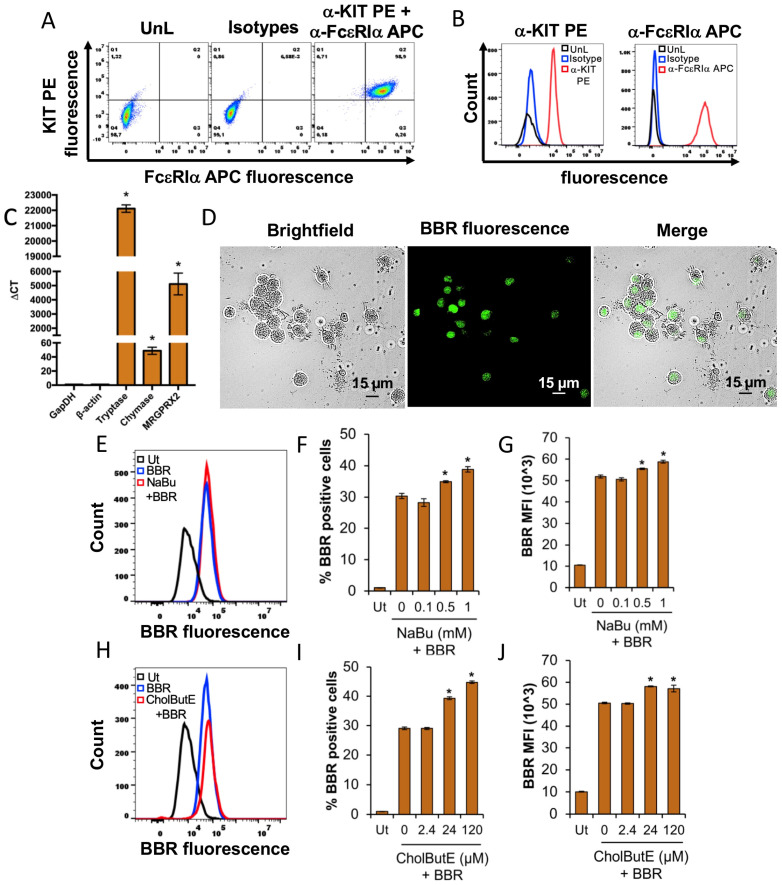
Sodium butyrate (NaBu) and cholesteryl butyrate emulsion (CholButE) modestly increase BBR fluorescence in LAD2. (**A**) A total of 100,000 LAD2 cells in PBS/BSA buffer were immunolabeled with α-KIT PE+ α-FcεRIα APC antibodies or the corresponding isotype controls for 1 h at 4 °C in the dark. The HMC-1.2 cells were washed 3× with the PBS/BSA buffer and processed for flow cytometry. UnL represents unlabeled cells. In total, 20,000 cells were analyzed to determine (**A**) KIT PE versus FcεRIα APC fluorescence dot plots or (**B**) histogram overlay showing KIT PE or FcεRIα APC fluorescence of UnL isotype or α-KIT PE+ α-FcεRIα APC immunolabeled LAD2. The data shown in (**A**,**B**) are representative of three independent experiments. (**C**) The qRT-PCR analysis of human *TPSAB1 (Tryptase)*, *CMA1 (Chymase)*, and *MRGPRX2* mRNA expression in LAD2. *GapDH* and *β-actin* were utilized as endogenous controls to normalize the samples. The data from the three independent cultures is represented as average delta CT (critical threshold). A Student’s *t*-test was conducted to calculate the statistical significance (*p* < 0.05 = *) relative to *GapDH* and *β-actin*. (**D**) A total of 100,000 LAD2 cells were stained with 5 µM BBR for 24 h and analyzed under the Echo Revolve 4 hybrid fluorescence microscope at 20× magnification. The scale represents 15 µm. A total of 100,000 LAD2 cells were incubated with 0, 0.1, 0.5, or 1 mM NaBu (**E**–**G**) or 0, 0.10, 2.4, 24, or 120 µM CholButE (**H**–**J**) for 48 h followed by treatment with 5 µM BBR for 24 h. Ut represents the LAD2 cells that were treated with PBS. In total, 20,000 cells were analyzed by flow cytometry to determine (**E**,**H**) BBR fluorescence histogram, (**F**,**I**) % BBR positive cells, or (**G**,**J**) BBR mean fluorescence intensity (MFI). n = 3; a Student’s *t*-test was performed to determine the statistical significance (*p* < 0.05 = *) relative to 0 mM NaBu+BBR (**F**,**G**) or 0 µM CholButE+BBR (**I**,**J**). The data shown in (**E**,**H**) are representative of three independent experiments.

The results shown thus far suggested that NaBu and CholButE increased berberine fluorescence in HMC-1.2. We tested this possibility in a different human mast cell line LAD2 that expresses FcεRI and KIT receptors (Figure 10A,B). It also expresses the *Mas-related G protein-coupled receptor* (*MRGPRX2*), *TPSAB1* (*Tryptase*), and *CMA1* (*Chymase*) mRNA (Figure 10C). Similar to HMC-1.2, when the LAD2 cells were stained with 5 µM berberine for 24 h, berberine fluorescence was associated with the cytoplasm. However, unlike the HMC-1.2 cells where the fluorescence was localized to small punctate areas throughout the cytoplasm, berberine fluorescence in LAD2 coalesced into large regions, often to one side of the cell (Figure 10D).

To evaluate the effects of NaBu or CholButE on berberine fluorescence, LAD2 cells were incubated with NaBu (0, 0.1, 0.5, or 1 mM) or CholButE (0, 2.4, 24, or 120 µM) for 48 h followed by treatment with 5 µM berberine for 24 h. The flow cytometry results show that NaBu (Figure 10E–G) and CholButE (Figure 10H–J) modestly increase berberine fluorescence as indicated by a shift of the berberine fluorescence histogram towards the right (Figure 10E and Figure 10H, respectively), and an increase in the percent of berberine positive cells (Figure 10F and Figure 10I, respectively) and berberine MFI (Figure 10G and Figure 10J, respectively).

## 4. Discussion

Although the role of butyrate and GAGs such as heparin is important in gut health, the relationship between butyrate, mast cells, and the mast cell production of GAGs is not well understood. Therefore, we examined the effect of butyrate on human mast cell heparin production and storage.

We have previously shown that berberine is internalized by mast cells in an energy-dependent process and interacts with intracellular granules which contain GAGs such as heparin [39,40]. Furthermore, berberine specifically interacts with heparin and thus, changes in berberine fluorescence can be used as a reasonable indicator of changes in heparin content [30,31]. Our NMR analysis showed that berberine interacts with heparin and berberine staining can be used as a reliable method of heparin detection. Based on our NMR analysis, we can conclude that berberine would have a similar interaction with other GAGs that have a similar structure to heparin. Therefore, although we have focused our conclusions on heparin, it is possible that berberine is also binding to other GAGs in the mast cell granules.

Our biochemical analysis indicated that NaBu treatment increased berberine fluorescence, suggesting that GAGs within HMC-1.2 increased in a time- and concentration-dependent manner. Moreover, NaBu-mediated increase in berberine fluorescence required 48–72 h for optimal effect since an increase in berberine fluorescence was not observed when HMC-1.2 cells were incubated with NaBu for 24 h. This is most likely because butyrate effects require several steps: NaBu must first penetrate the cells, inhibit the HDACs [4] in the nucleus, and then modify gene transcription and protein translation. To support the hypothesis that NaBu increases GAG content, we show that 1 mM NaBu treatment increased HMC-1.2 granularity. However, we were unable to measure the total GAG content present within HMC-1.2 lysates using a commercially available kit based on competitive ELISA, and further optimization is required to ascertain if total GAGs could be detected using ELISA. Furthermore, HPLC analysis showed that 1 mM NaBu increases heparin content within HMC-1.2 lysates. Moreover, NaBu treatment did not proportionately increase the staining of HMC-1.2 with an amine-reactive near-IR dead cell fluorescent dye, suggesting that the cell membrane remained intact and an increase in berberine fluorescence was observed specifically upon NaBu treatment.

Butyrate is an HDACi and it has been shown that NaBu effects on mouse and human mast cells function are partly mediated by the epigenetic modification of specific genes such as FcεRI [9]. To determine whether NaBu was similarly changing the expression of enzymes associated with GAG synthesis, we measured the mRNA expression of genes involved in heparin/heparan sulfate biosynthesis such as *GLCE*, *NDST1*, *NDST2*, *HS6ST1*, and *GALT1*. Our results show that NaBu increased the expression of *GLCE*, *NDST1*, *NDST2*, *HS6ST1*, and *GALT1* in a time-dependent manner with the most prominent effects observed 72 h post treatment. These results are further supported by our HPLC analysis where we observed a greater increase in heparin content at 72 h post 1 mM NaBu treatment as compared to 48 h. The expression of *NDST1*, *NDST2*, and *HS6ST1* was increased 4- to 6-fold which was the highest among all of the genes that were tested. These results support the hypothesis that an increase in HMC-1.2 granularity upon 1 mM NaBu treatment and heparin content may be dependent upon an increase in the expression of GAG biosynthesis enzymes. These increased heparin biosynthesis enzymes produce more heparin which results in an increase in berberine fluorescence. However, it is possible, but less likely, that berberine could have a direct effect on altering the expression of the genes involved in the heparin biosynthesis pathway.

NaBu is a sodium salt of butyric acid and when dissolved in water, it dissociates into butyrate. Some of the butyrate ions react with water to form butyric acid and hydroxide ions. These varying forms of butyrate and butyric acid have been shown to have varying effects in the gut of animals and specifically on the growth of bacteria [41]. Butyrate nanoemulsions, usually composed of cholesterol and other helper lipids, have been developed to increase the delivery and efficacy of butyrate, particularly in the gut due to their small droplet size and stability [42,43,44]. To determine whether such an emulsion would have similar effects as NaBu, we synthesized a CholButE which modifies the proliferation of cells [26]. Our data showed that 240 µM and 1000 µM CholButE were toxic to HMC-1.2, but 120 µM and 24 µM CholButE did not affect HMC-1.2 viability. These biocompatible concentrations of CholButE increased berberine fluorescence in a time- and concentration-dependent manner, suggesting that they increased the GAG content in these cells. Furthermore, the flow cytometry analysis indicated that 120 µM CholButE slightly increased the granularity of the HMC-1.2 as indicated by an increase in SSC.

Furthermore, CholButE reduced HMC-1.2 proliferation in a time- and concentration-dependent manner without substantially affecting cell viability or metabolic activity as previously published by our lab for NaBu [12]. These results collectively suggest that CholButE reduces HMC-1.2 proliferation but slightly increases granularity as indicated by an increase in berberine fluorescence, causing them to become more differentiated into a mature mast cell phenotype.

In contrast to the gene expression data with NaBu, 120 µM CholButE did not appreciably alter the mRNA expression of *GLCE*, *NDST1*, *NDST2*, *HS6ST1*, and *GALT1* which suggests that the slight changes in granularity corresponded to only minor changes in GAG biosynthetic enzyme expression. The increase in mRNA expression upon the CholButE treatment was not as high as observed with NaBu (1.4-fold versus 6-fold), where a greater effect of NaBu was observed with HMC-1.2 granularity. The different effects of NaBu versus CholButE could be explained by several possibilities. First, we used a lower concentration of CholButE (120 µM) in our experiments than NaBu (1 mM) since 1 mM CholButE was toxic to HMC-1.2. Second, NaBu dissociates into butyrate whereas cholesteryl butyrate is a derivative that has different stability and solubility. Cholesteryl butyrate is formed when butyrate is esterified with cholesterol, resulting in a compound that combines the properties of both cholesterol and butyrate. This esterification alters the molecule’s solubility and its interaction with biological membranes, potentially affecting its absorption and function in the body. Therefore, it is likely that NaBu is more effective in crossing cellular membranes, entering the nucleus, and altering genes involved in GAG synthesis compared to CholButE. Moreover, CholButE appears to reduce the mRNA expression of certain genes at 72 h. It is possible that CholButE may alter GAG content by targeting a different biosynthetic pathway.

Nevertheless, CholButE did not increase the fluorescence of other non-GAG binding dyes such as calcein acetoxymethyl, propidium iodide, Nile red, or free amine-reactive near-IR dead cell stain suggesting that the CholButE effect of increasing berberine fluorescence was specific to a parameter that was associated with berberine. Given that berberine interacts with heparin, it is reasonable to assume that both NaBu and CholButE increase heparin content in HMC-1.2 which is a unique observation since no other compound has been shown to increase heparin production and storage in mast cells. To our knowledge, other mediators and cytokines that are known to promote mast cell differentiation (stem cell factor, interleukin-3, interleukin-9, and interleukin-4) have not been demonstrated to increase heparin production in either human or rodent mast cells. Therefore, this would be a unique mechanism for promoting mast cell granulation—particularly in the gut where butyrate levels are high and mast cells are present in significant numbers in the submucosa. This speculation is backed up by our LAD2 data where only a modest effect of NaBu on berberine staining was observed. This could be due to several reasons such as (1) a slower growth rate of LAD2 (approximately 10 days doubling time) compared to the HMC-1.2 (approximately 48 h doubling time); and/or (2) the differences in the phenotype of these two mast cell models. The LAD2 cells express FcεRI, MRGPRX2, chymase, and tryptase which demonstrates the “connective tissue phenotype”. However, HMC-1.2 cells do not express FcεRI, MRGPRX2, or chymase, demonstrating their “mucosal phenotype”. The mast cells in the gut submucosa would be more akin to the HMC-1.2 phenotype. Therefore, it is possible that the effect of NaBu is specific to the submucosal mast cell phenotype.

## 5. Conclusions

**Figure 11:**
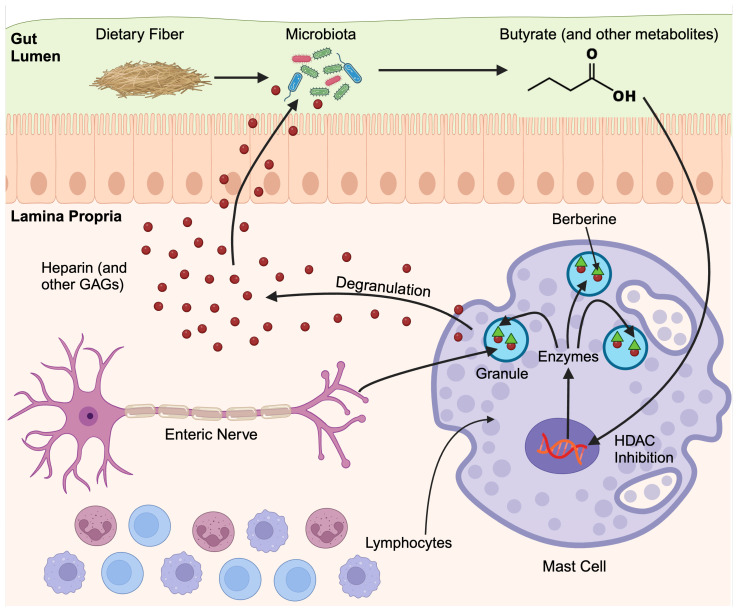
The role of mast cells in regulating GAG production in response to butyrate in the gut mucosa. In this hypothetical model, commensal gut microbes produce butyrate in response to fiber. The butyrate could promote the production of GAGs in mast cells by the epigenetic modification of genes encoding enzymes responsible for GAG biosynthesis. Gut mast cells are located in the submucosa in the lamina propria and are closely associated with enteric nerves. GAGs released by gut mast cells, in response to signals from enteric nerves or other resident immune cells, could travel to the gut lumen and serve as an important carbon source, further supporting the growth of microbes.

Heparin is an important mediator in the gut [22]. Although it is used as an anticoagulant therapeutically, it is an important chemical in biotechnological and pharmaceutical processes and is produced in vast quantities commercially [45]. Heparin’s main purpose in normal physiological processes is probably unrelated to coagulation since heparin is conserved in invertebrates that do not have a blood coagulation system [46,47]. In fact, it has been suggested that heparin’s main role is to limit neoplastic activity, and could be used to treat inflammation, infertility, and infectious disease [19,47,48,49]. Our data presents the intriguing hypothesis that butyrate produced by gut bacteria could induce intestinal mast cells to produce heparin which, in turn, could regulate gut microbiota (Figure 11). Therefore, this could represent an interesting host–microbiota communication conduit that facilitates gut homeostasis. Certainly, mice that do not express heparin in intestinal mast cells show increased gut inflammation and an increased expression of the important mast cell activation receptor mas-related G protein-coupled receptor (MrgpB2), suggesting that heparin produced by mast cells is an important regulatory mediator in the gut [50].

Conversely, our data with CholButE suggest that such mixtures may be effective treatments to maintain gut homeostasis. Other, similar forms of butyrate such as cholesteryl butyrate solid lipid nanoparticles (CholButSLNPs) can inhibit the adhesion, migration, and proliferation of colon cancer cells [26,51] suggesting that they can have some beneficial effects in the gut. The coadministration of dexamethasone with CholButSLNP has anti-inflammatory effects against inflammatory bowel disease [52,53]. Several preclinical and clinical studies have shown that butyrate helps maintain intestinal homeostasis and modulates gut health [54]. However, it has not been determined whether these cholesterol butyrate formulations have any effects on the gut microflora and it remains an interesting new avenue of investigation.

## Figures and Tables

**Table 1:** List of introns spanning oligonucleotide primers used in this study.

Gene ID	Name (Abbreviation)	Forward Primer	Reverse Primer
AB028600.1	Homo sapiens galactosyltransferase I (GALT1)	CTGAACAGGACAACCTCTCATC	CAGAAGATCTGGCAACACTAGG
NM_001324094.2	Homo sapiens glucuronic acid epimerase (GLCE)	TGCCAAAGGGCTGCTTTA	ACACCTTCACTGGTTTCTGG
NM_001543.5	Homo sapiens N-deacetylase and N-sulfotransferase 1 (NDST1)	GACGTGAAGGCCCTGTTT	TGGTACCTGTGTGGAAGAATTT
NM_003635.4	Homo sapiens N-deacetylase and N-sulfotransferase 2 (NDST2)	CCGCTACATCTTGGTAGACATC	TCAACAGAGCCTCAACATCAG
NM_004807.3	Homo sapiens heparan sulfate 6-O-sulfotransferase 1 (HS6ST1)	ACGCCCAGGAAGTTCTACTA	GTGCGCCCATCACACATA

**Table 2:** Chemical shifts of 1 mM berberine in the absence and presence of 5 mg/mL heparin. Note that the maximum chemical shift change [Δδ (ppm)] is observed with H13 and H1 shown in yellow and red, respectively.

δ (ppm)	H8	H13	H11	H12	H1	H4	-OCH2O-	H6	9-OCH3	10-OCH3	H5
Absence	9.719	8.649	8.13	8.04	7.628	7.005	6.126	4.912	4.17	4.127	3.268
Presence	9.683	8.549	8.08	7.99	7.546	6.977	6.108	4.899	4.159	4.099	3.255
Δδ (ppm)	0.036	0.1	0.05	0.05	0.082	0.028	0.018	0.013	0.011	0.028	0.013

## Data Availability

The original contributions presented in the study are included in the article/Supplementary Materials; further inquiries can be directed to the corresponding author.

## Supplementary Materials

The following supporting information can be downloaded at: https://www.mdpi.com/article/10.3390/cells13151241/s1, Figure S1: (A,B) 100,000 HMC-1.2 were incubated with 1 mM NaBu for 24 h followed by treatment with 5 µM BBR for 24 h. Cells were collected and processed for flow cytometry to visualize (A) side scatter (SSC) vs forward scatter (FSC) or (B) BBR fluorescence histogram. HMC-1.2 cells were treated with 1 mM NaBu followed by measuring cell viability using trypan blue exclusion assay (C) and metabolic activity using XTT assay (D) at 24, 48 and 72 h. Ut represents “untreated cells”. n = 3–7. A Student’s *t*-test was performed to determine statistical significance (*p* < 0.05 = *) relative to Ut samples of corresponding time points. Data shown in “A” and “B” is representative of three independent experiment; Figure S2: (A–C) 100,000 HMC-1.2 were treated with 0, 2.4, 24 or 120 µM CholButE for 24 h followed by treatment with 5 µM BBR for 24 h and processed for flow cytometry to determine (A) SSC vs BBR fluorescence dot plots, (B) % BBR positive cells or (C) BBR MFI. Ut represents “untreated cells”. n = 3, a Student’s *t*-test was performed to determine statistical significance (*p* < 0.05 = *) relative to 0 µM CholButE + BBR in B and C. Data shown in “A” is representative of three independent experiment; Figure S3: CholButE-mediated increase in fluorescence is specific to BBR: (A) 100,000 HMC-1.2 were incubated with 120 µM CholButE for 24 h followed by treatment with 50 µM BBR for 3 h. Cells were collected and processed for flow cytometry to visualize BBR fluorescence histogram (A-i) and SSC vs BBR fluorescence dot plot (A-ii). Data was analyzed relative to untreated HMC-1.2 that has been treated with 50 µM BBR for 3 h. Data presented is representative of three independent experiments.
