# Supplementary material for: Butyrate Increases Heparin Synthesis and Storage in Human Mast Cells
Source: Cells. 2024 Jul 24;13(15):1241. doi: 10.3390/cells13151241 (PMC11311861; doi:10.3390/cells13151241)
Supplement: Supplementary file 1 [file cells-13-01241-s001.zip › cells-3037140-supplementary.pdf]

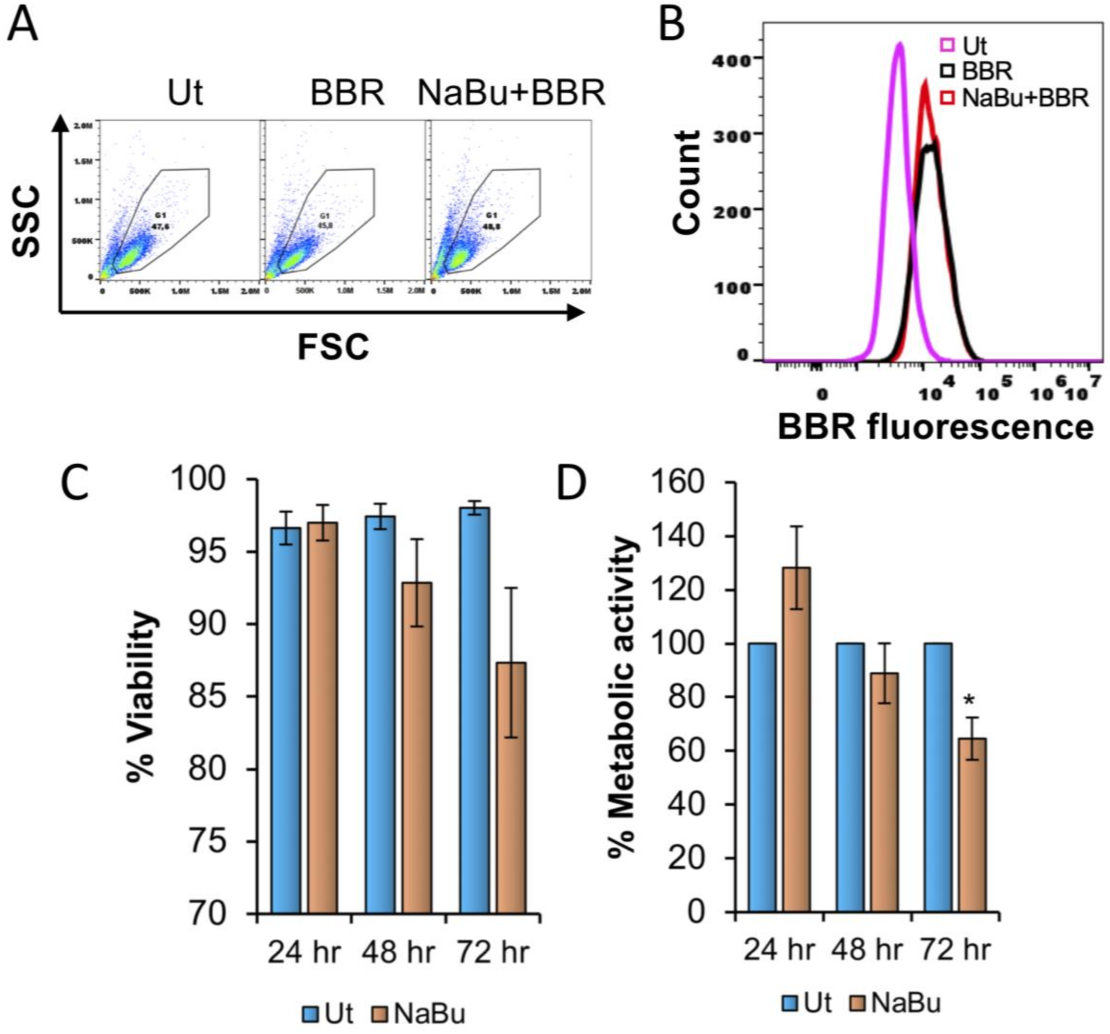

Supplementary Figure S1: (A-B) 100,000 HMC-1.2 were incubated with 1 mM NaBu for 24 h followed by treatment with 5 µM BBR for 24 h. Cells were collected and processed for flow cytometry to visualize (A) side scatter (SSC) vs forward scatter (FSC) or (B) BBR fluorescence histogram. HMC-1.2 cells were treated with 1 mM NaBu followed by measuring cell viability using trypan blue exclusion assay (C) and metabolic activity using XTT assay (D) at 24, 48 and 72 hr. Ut represents "untreated cells". n=3-7. A Student's t-test was performed to determine statistical significance (p<0.05=*) relative to Ut samples of corresponding time points. Data shown in "A" and "B" is representative of three independent experiment.

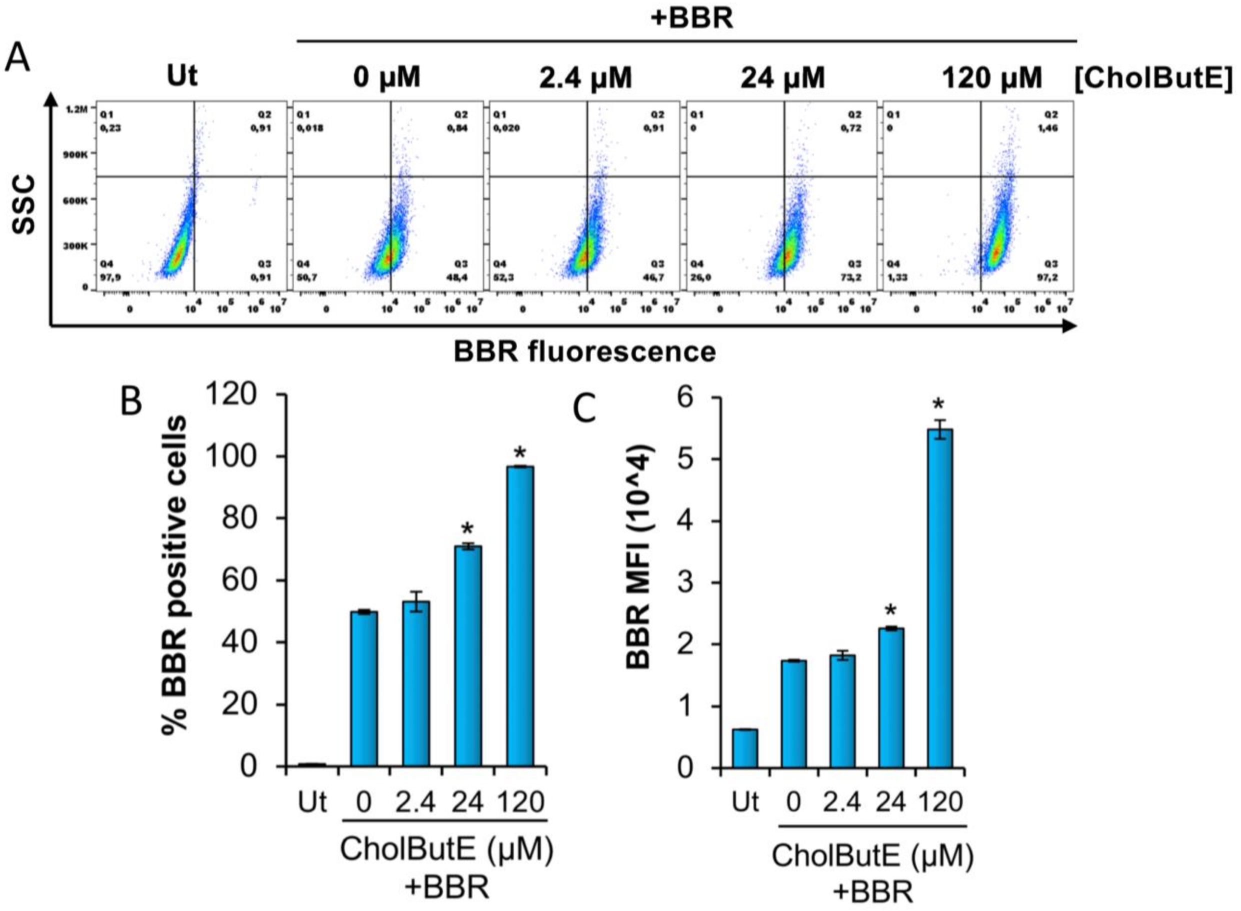

Supplementary Figure S2: (A-C) 100,000 HMC-1.2 were treated with 0, 2.4, 24 or 120 µM CholButE for 24 h followed by treatment with 5 µM BBR for 24 h and processed for flow cytometry to determine (A) SSC vs BBR fluorescence dot plots, (B) % BBR positive cells or (C) BBR MFI. Ut represents "untreated cells". n=3, a Student's t-test was performed to determine statistical significance (p<0.05=*) relative to 0 µM CholButE + BBR in B and C. Data shown in "A" is representative of three independent experiment.

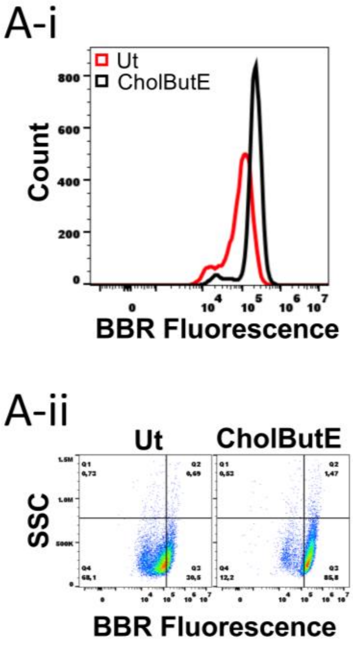

Supp Figure S3: CholButE-mediated increase in fluorescence is specific to BBR: (A) 100,000 HMC-1.2 were incubated with 120 µM CholButE for 24 h followed by treatment with 50 µM BBR for 3 h. Cells were collected and processed for flow cytometry to visualize BBR fluorescence histogram (A-i) and SSC vs BBR fluorescence dot plot (A-ii). Data was analyzed relative to untreated HMC-1.2 that has been treated with 50 µM BBR for 3 hr. Data presented is representative of three independent experiments.
